# Facilitating the design of HL7 domain models through a model-driven solution

**DOI:** 10.1186/s12911-020-1093-4

**Published:** 2020-05-25

**Authors:** M. A. Olivero, F. J. Domínguez-Mayo, C. L. Parra-Calderón, M. J. Escalona, A. Martínez-García

**Affiliations:** 1 Web Engineering and Early Testing research group. Higher Technical School of Computer Engineering, University of Seville, Seville, Spain; 2 Istituto di Scienza e Tecnologie dell’Informazione, Consiglio Nazionale delle Ricerche, Pisa, Italy; 3 Computer Languages and Systems Department, University of Seville, Seville, Spain; 4 Group of Research and Innovation in Biomedical Computing, Biomedical Engineering and Health Economics, Institute of Biomedicine of Seville, IBiS / Virgen del Rocío University Hospital / CSIC / University of Seville, Seville, Spain

**Keywords:** UML, HL7, MDE, Domain models, Metamodel, MoDHE

## Abstract

**Background and goal:**

Health information systems are increasingly sophisticated and developing them is a challenge for software developers. Software engineers usually make use of UML as a standard model language that allows defining health information system entities and their relations. However, working with health system requires learning HL7 standards, that defines and manages standards related to health information systems. HL7 standards are varied, however this work focusses on v2 and v3 since these are the most used one on the area that this work is being conducted. This works aims to allow modeling HL7 standard by using UML.

**Methods:**

Several techniques based on the MDE (Model-Driven Engineering) paradigm have been used to cope with it.

**Results:**

A useful reference framework, reducing final users learning curve and allowing modeling maintainable and easy-going health information systems.

**Conclusions:**

By using this approach, a software engineer without any previous knowledge about HL7 would be able to solve the problem of modeling HL7-based health information systems. Reducing the learning curve when working in projects that need HL7 standards.

## Introduction

Nowadays, health information systems are increasingly sophisticated [1]. The development of high-quality, maintainable and interoperable products is a challenge for software developers that compete in finding a niche market within the health systems field. The need of working with an electronic health record shared all over the word is a fact today [2]. For this purpose, it is essential to use health information standards that allow establishing health information exchange rules [3]. However, engineers are not always proficient with health standards. The initial hypothesis is that since software engineers can read UML (Unified Modeling Language) [4] but are not usually proficient with HL7, having a UML translation of HL7 will reduce the learning curve and thus easing the adoption of this health standard. To validate this hypothesis, this work aims to bring closer health standards and software definition standards.

Software engineers usually work by using UML to define the projects. UML is a standard model language proposed by the OMG (Object Management Group). This organization promotes the use of object-oriented technologies through the creation and preservation of guidelines, standards, and specifications. MDE (Model-Driven Engineering) is a paradigm that focuses on the creation and operation of domain models. A domain model is a conceptual model that describes entities, attributes, roles, relations and restrictions associated with the domain of the problem. It describes concepts dealing with the nature of the problem, instead of describing concepts related to software systems. It helps software engineers to decouple representation by focusing on the concepts. A metamodel is a model that describes the concepts used in a specific domain model [5, 6]. MDE is used in this study since this paradigm has been used successfully in many other research topics e.g. business process management [7], and in software testing area [8, 9] among others.

Many notations exist for standing for metamodels, UML being one of the most frequently used.

In this way, this work is referring to UML class-diagram modeling notations.

On the other hand, HL7 International (Health Level Seven International, from now on it will appear as “HL7”) is a non-profit international organization that promotes and defines standards related to health information systems. The members of this organization develop standards with the purpose of allowing exchange and integrate the electronic health record with the aim of supporting clinical practice as well as management, development and evaluation of health services.

HL7 counts on 31 affiliate countries; Spain, for instance, is included through the organization named HL7 Spain. Currently, HL7 is offering three certifications that validates the knowledge about HL7 standards and it comprises nearly 4800 validated researchers all over the world.

This information reinforces the substantial impact that HL7 has internationally, considering that there is a considerable amount of worldwide cases of implementations based on HL7 standards [10].

This organization defines domain models for each HL7 standard for the whole patient data, with the aim of representing each problem or working scenario identified over time.

Most HL7 standards have a common metamodel called MIF (Model Interchange Format) [11]. Initially, MIF was created to represent the HL7 v3 metamodel using UML, with the aim of leverage MDE benefits, and address some shortcomings in the UML paradigm itself. Most HL7 domain models could be modeled following this metamodel, which is formally modeled with one of the HL7 standards [12]. It should be pointed out that MIF is so large, and it is presented in such an abstract way that, although it seems very interesting from a conceptual point of view, it could cause lots of difficulties when using and learning it.

Each HL7 standard has an underlying domain analysis model that specializes and extends MIF. In this research, we are focusing in HL7 v2 and HL7 v3 since these are the models being used in the area on which it is intended to be validated this approach. Each HL7 domain analysis model was understood in some cases as a metamodel, since such models can be used to instantiate implementation guides covering a specific scenario. In some cases, these models have not been explicitly defined in a diagram, but in a text on different documents. In some other cases, these models have been explicitly defined in diagrams that use HL7’s own graphical language. Keeping in mind that models of some HL7 standards are described by means of a text in large documents, and other HL7 standards are modeled using its own graphical language, it is not easy for a software engineer to design a domain model of a software solution according to a specific HL7 standard. In contrast to what happens when using HL7 standards, software engineers usually feel conformable with general model languages, such as UML.

Therefore, in this research work we have been carrying out the research under the hypothesis of providing software engineers a solution that allows them to design their domain models proposals to develop health information systems by means of systematically using UML notation. Thus, in a transparent way, these representations could take part in health information systems that are conform to HL7. For this purpose, we propose the use of the MDE paradigm.

In conclusion, along this study we will offer a solution consisting in using MDE to solve the problem of designing domain models according to HL7, using an UML-based interface to reduce the learning curve and cost that this problem involves.

Up to now, in this research, the effort has been focused on three HL7 standards: HL7 v3, HL7 CDA and HL7 v2.x. Future work will include an evaluation of the results of the validation and further models of HL7 being included on this framework.

In addition, MDE text transformations can extend this work to allow MoDHE model to automatically generate code compliant with technical interoperability standards as FHIR.

This paper is structured as follows. After this introduction, Section 2 describes the background together with the problem and justifies the proposed solution. Then, Sections 3 and 4 explain the methodology used and the results obtained, respectively. To finish, Section 5 supplies further discussion and Section 6 states final conclusions.

## Background

HL7 international is an organization that provides standards in the health context. These standards can use health data safely, whenever, and wherever needed. This organization uses information models to define standards. Most of these models extend MIF which is the metamodel of HL7 models. So, each HL7 model is conform to MIF definitions.

Although HL7 is an organization that places strong emphasis on interoperability standards, most of the HL7 V3 standards have been designed using a language with graphical code specific to HL7. Lots of researchers from the scientific community have identified the need of using HL7 in the MDE context to have model support tools or testing tools among others at their disposal [13–16].

Therefore, this work aims to find solutions to the following problems:

- Learning curve. UML is a standard to model well known and used software systems. Most of HL7 models are designed following their own graphical model language, which is known and used by HL7 members and software engineers who eventually have worked with HL7 standards. Some HL7 models are not modeled in a graphical way, but in texts and large documents. Therefore, software engineers, face up a bigger learning curve at designing a health system based on HL7 than designing an UML domain model.
- Usability. Tools that allow working with HL7 standards are limited and less usable than tools that use a standardized model language, as they explicitly use the semantics of HL7. On the other hand, there are lots of tools to design systems using UML, which are more usable because the software engineer is more familiar with their semantics. If an engineer designs a healthcare computing system using of a tool that conforms to both UML elements and HL7 elements in a straightforward way, the usability of the tool will increase.
- Maintenance and adaptation. Considering the high complexity and size of HL7 standards, healthcare systems created according to HL7 are less maintainable and have less adaptation capacity than those common systems that are not from the healthcare environment. However, if we evaluate the maintenance and adaptation capacity of an UML-based information system, we will deduce that the system has those capacities, since UML is a model language extensively known by software engineers.

Consequently, the need of providing software engineers with a reference framework that let them approach these two standards is shown in this solution: UML general-purpose model language and HL7 standards, through the MDE paradigm.

There are previous identified experiences regarding the use of HL7 standards that applied techniques based on the MDE paradigm [17–20]. For years, they highlighted the importance of model information in the health context for sharing knowledge, improving processes and document the requirements of software solutions for health information systems. Although these experiences use MDE and HL7, an obstacle was mainly identified: even though they used techniques based on MDE (e.g. models transformation or mechanisms for automatically generate code), they did not use HL7 completely. Authors in these works addressed the research to a single specific HL7 model, keeping others out of the scope of their research.

The aim of this solution is to back up the development of health information systems based on HL7, through a reference framework that brings closer HL7 standards and UML general-purpose model language to the developers, using the MDE paradigm.

NDT (Navigational Development Techniques) is a methodology based on MDE that offers formal and complete support to the software lifecycle (viability study, requirements, analysis, design, implementation, maintenance and test) [21]. NDT methodology covers the phases of the lifecycle in a structured way, reducing errors and redundancies. This methodology defines a set of metamodels (and transformations among them) to formally address the phases of the lifecycle of a software product or service. Up today, NDT has been applied to many projects and business experiences [22, 23] However, NDT is a software development methodology for general purposes and it doesn’t included mechanisms to define interoperability requirements, as the one based in HL7 standard, in this way, NDT has been extended to offer this feature.

**Fig. 1:**
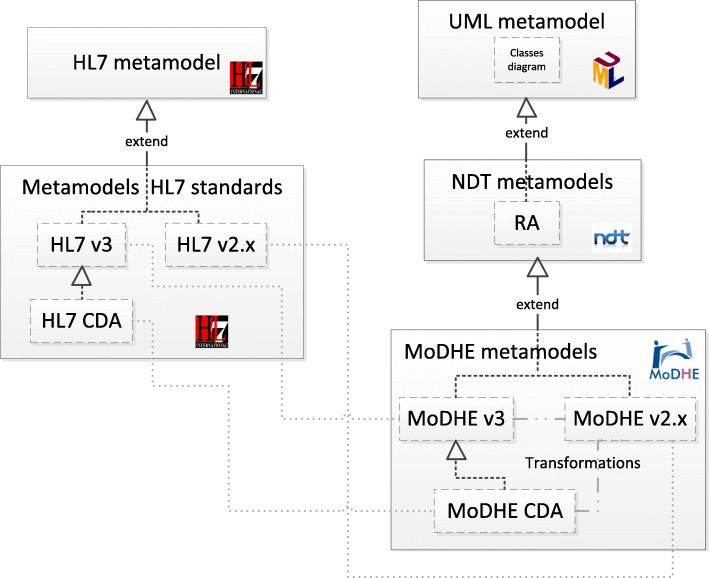
Formal definition of the proposal

Figure 1 shows graphically the proposed solution. On one hand, HL7 metamodels are represented on the left. On the other hand, our approach, named MoDHE, MOdel-Driven Health Engineering, and its metamodels are displayed on the right extending NDT metamodels. Specifically, we extend Information Requirements, (RA) metamodel in NDT methodology, which subsequently extend from UML metamodels. In particular, it extends the class diagram metamodel. Derivation mechanisms are also shown among MoDHE metamodels. Both, HL7 metamodels and MoDHE metamodels establish relations one to one, since it is necessary to consider metamodels and constraints of HL7 standards to define MoDHE metamodels.

This reference model has been called MoDHE due to the close relation with the MDE paradigm and the health context.

## Methods

The current research line contributes to the academic field with a reference framework composed of three basic pillars that offer the following added value to the existent scientific base. Our methodology. It is a procedure to design HL7 domain models in a health area software project with an UML-based interface. The HL7-based model language, that is the language that extends UML for modelling health information systems by HL7 standards. And derivation mechanisms. Key element to enhance interoperability among standards and ease systems maintenance and extension.

**Fig. 2:**
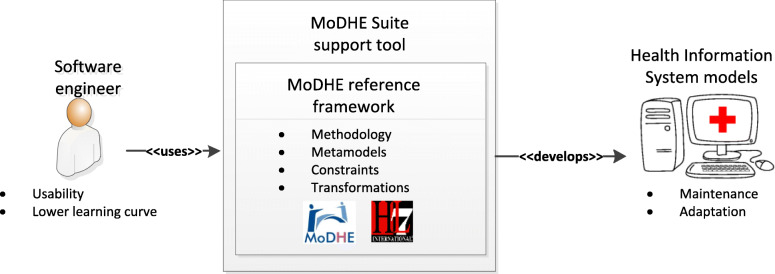
Context

Figure 2 offers a view of the context where MODHE reference framework is developed.

### Methodology

Initially, after reviewing the correspondences between HL7 and UML metamodel in previous works [24, 25], the authors started working on MDE techniques to implement Model-to-Model techniques to generate HL7 models automatically, by using UML models as a source [26]. Afterwards, bearing in mind that software engineers, who model health software, must model artifacts in all the phases of the software lifecycle as non-health software; the authors decided to develop MoDHE methodology as an extension of NDT methodology. Thus, authors decided to extend the metamodels that covered the elements related to the software lifecycle and enrich NDT by adding the metamodels of HL7 standards through the defined DSL, Domain Specific Language, that works out UML-like notations for HL7 concepts. Therefore, they got a formal and complete framework that enabled modeling a health information system systematically according to HL7, using class diagrams models.

In consequence, MoDHE methodology helps software engineers model requirements using an UML language, by defining HL7 requirements in a clear and systematic way.

MoDHE methodology extends the metamodels from the NDT phase called System Requirement Development (DRS). More specifically, it extends RA metamodel to include the elements of HL7 standards. Within RA metamodel, MoDHE methodology focuses on information requirements. Thank to this fact, a software engineer could define the health requirements catalog according to HL7.

The learning curve of a software engineer using MoDHE for the first time is minimum, because, like when using NDT, MoDHE methodology proposes to utilize UML class diagram notation to define models.

To develop MoDHE methodology, we analyzed in detail each metamodel (designed either by means of their own graphical model language or textual documents) for every HL7 standard that must be integrated, to get a deep knowledge about:

- The entities the metamodel includes.
- The constraints some entities must include to cover the definition of each entity.
- The transformation rules that could be identified among entities included by different standards.

This analysis was performed to get the required knowledge to model all the elements comprising MoDHE metamodels that, as mentioned before, constitute an extension of NDT metamodels.

Up to now, the effort has been focused on three HL7 standards that belong to software information requirements. These standards are also part of the DRS phase of NDT methodology. The effort has been focused on defining the static or structural phase of the system for HL7 v3, HL7 CDA and HL7 v2.x.

HL7 CDA standard is an extension of HL7 v3 standard, as Fig. 1 showed in section 1.

### HL7-based model language

MoDHE metamodel, a HL7-based model language, was created to support MoDHE methodology, as it was previously stated.

**Fig. 3:**
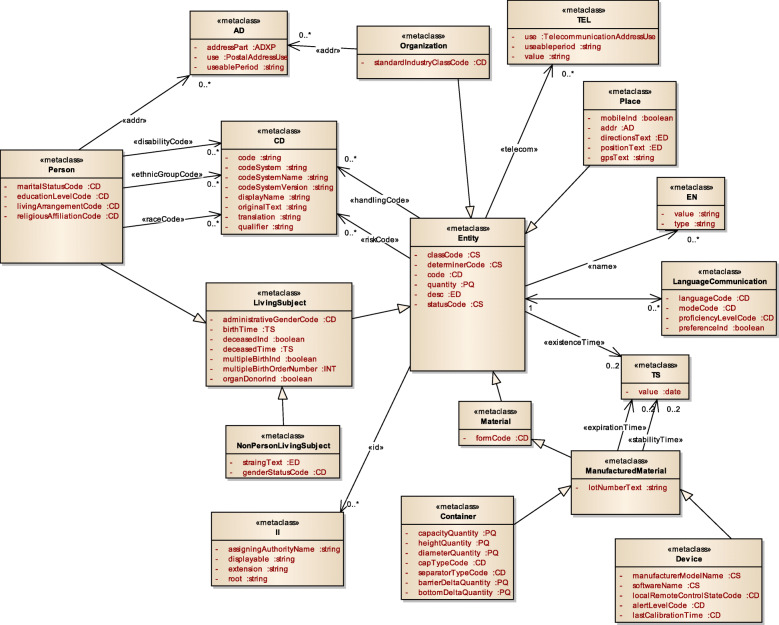
MoDHE metamodel

**Fig. 4:**
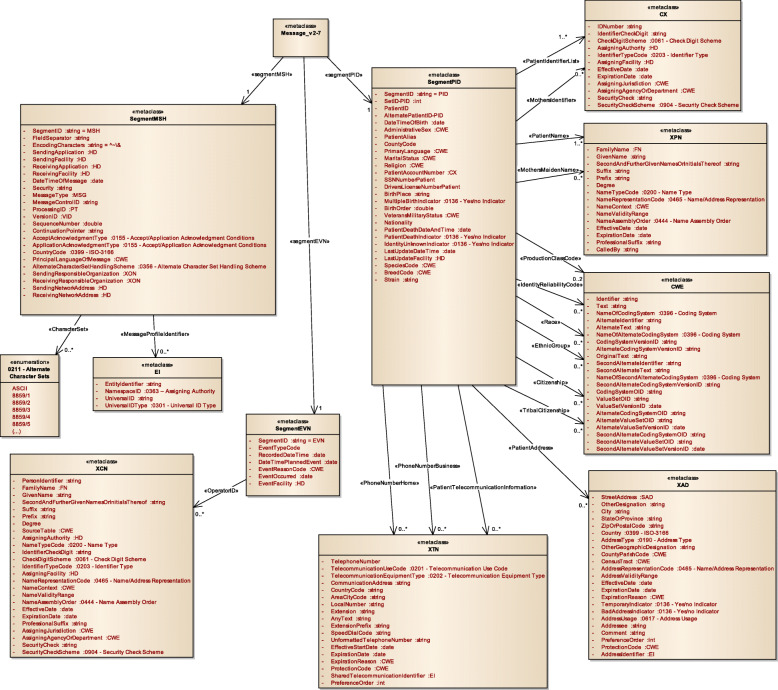
MoDHE metamodel for HL7 v2 entities

**Fig. 5:**
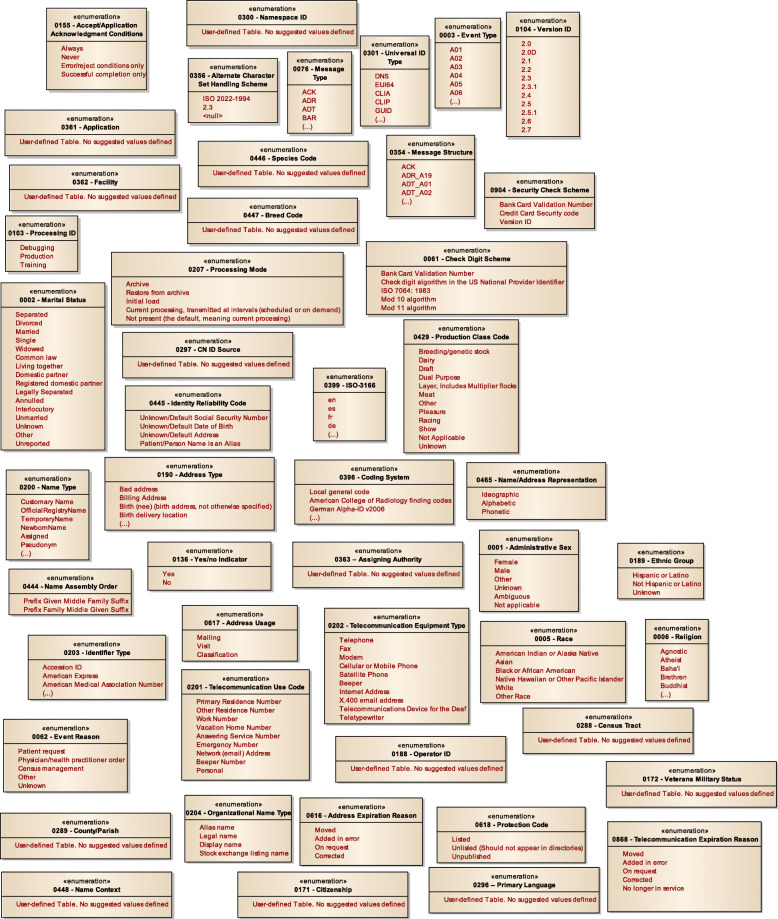
MoDHE metamodel for Hl7 v2 enumerates

**Fig. 6:**
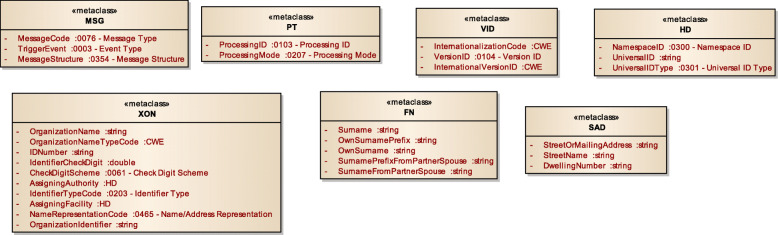
MoDHE metamodel HL7 v2 datatype

**Fig. 7:**
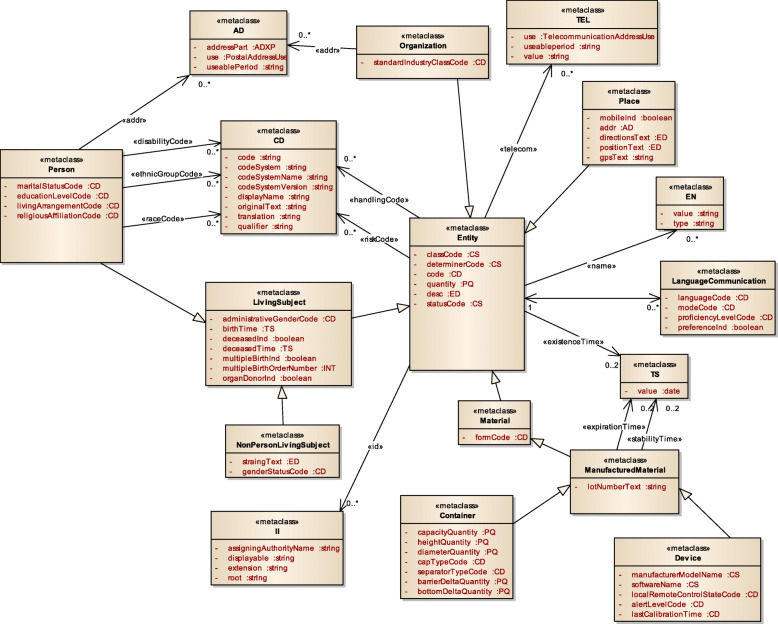
MoDHE metamodel HL7 v3 entities part 1

**Fig. 8:**
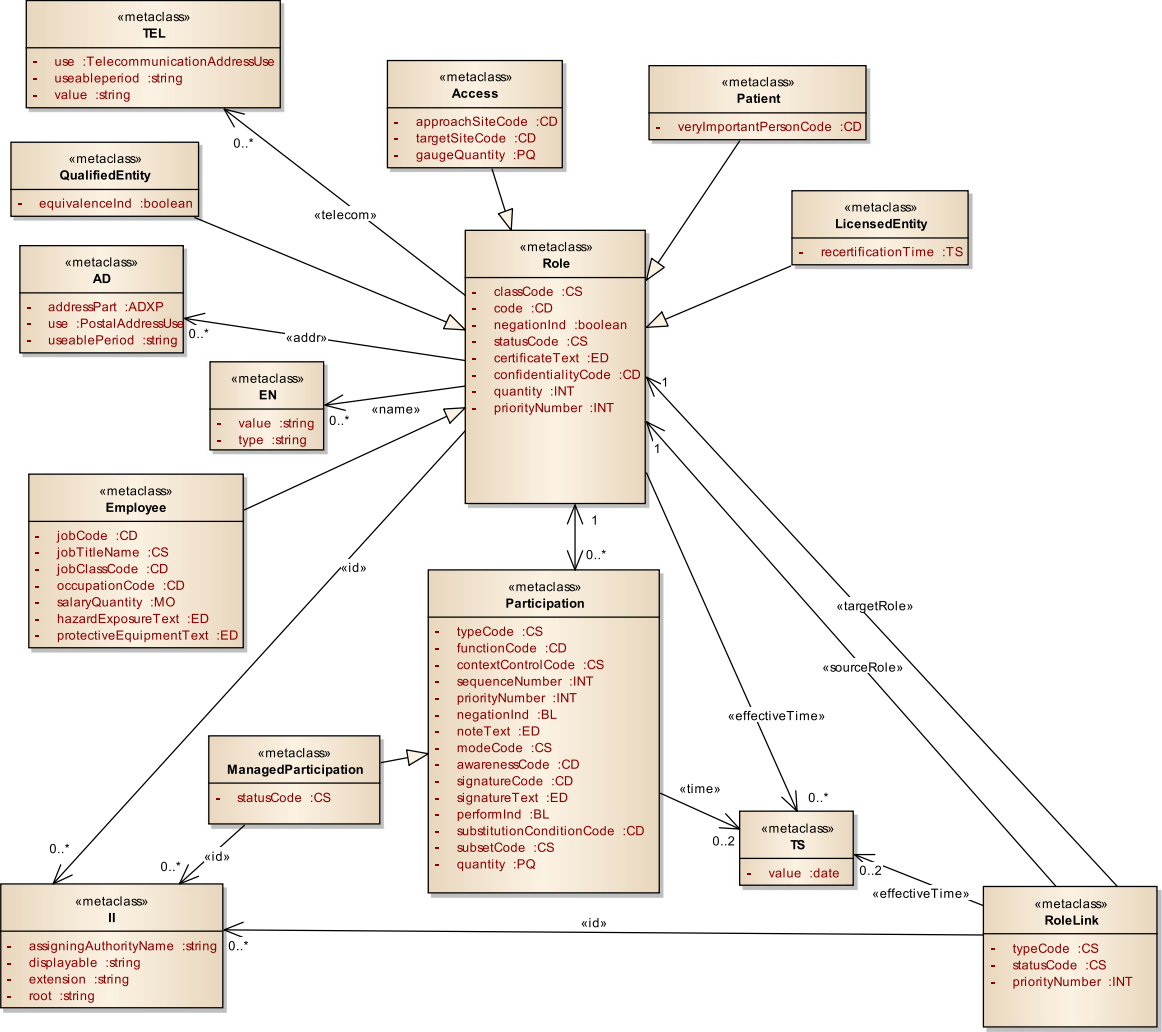
MoDHE metamodel HL7 v3 entities part 2

**Fig. 9:**
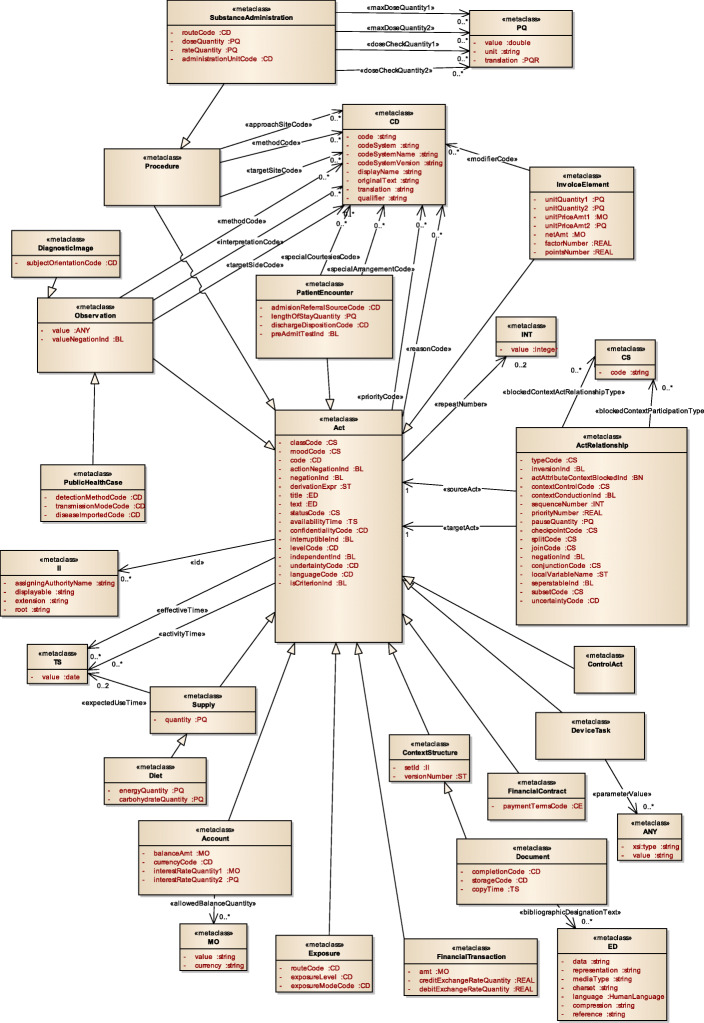
MoDHE metamodel HL7 v3 entities part 3

**Fig. 10:**
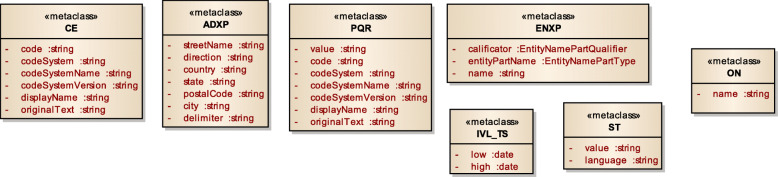
MoDHE metamodel HL7 v3 datatype

**Fig. 11:**
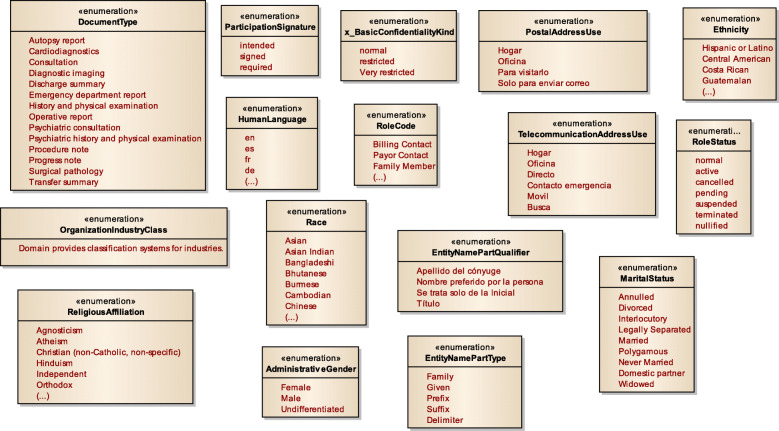
MoDHE metamodel HL7 v3 enum

Figure 3 displays a subset of MoDHE metamodels related to some entities of HL7 v3 standard. This metamodel extends the RA metamodel of NDT, which extends the Class model of UML, as depicted in Fig. 1. MoDHE metamodel has not been fully described since it is out of the scope of this paper and it would become too extensive, however Figs. 4, 5, 6, 7, 8, 9, 10, and 11 shows an overview of different metamodels that has been developed, so the reader can take a picture of MoDHE as a whole.

As Fig. 1 shows, MoDHE metamodels have been defined following the definitions and constraints included in HL7 standards.

### Derivation mechanisms

We can identify common elements when comparing reference models with the different HL7 standards. Such elements help defining easy transformations among entities from one standard into another one. Then, these elements enable creating partially the model structure, focused on a specific standard by taking a model based on a different standard as a reference. The systematic process to obtain a MoDHE model from another MoDHE model must consider the information from the source model and the transformation process that is applied (i.e., how this information is transformed, what dependencies among information elements exist, and what information is generated in the output model).

This process, entailing these semantic relations, establishes a transformation mechanism based on rules to obtain the final model. Thanks to this transformation process, a correspondence between both metamodels is set, so that the development process can be automated, and quality and consistency of models is improved. We have used QVT (Query/View/Transformation) language [27] to formally model these transformations. However, before copying with the formal definition of QVT transformation rules to derivate elements, it is necessary to identify the correspondence among elements in each metamodel.

As Fig. 1 displayed, some correspondences among HL7 standards have been identified.

As an example, Table 1 matches eight of these correspondences between HL7 CDA and HL7 v2.x standards.

**Table 1:** Correspondence among elements in the studied metamodels

HL7 CDA	HL7 v2.x	Field
ClinicalDocument.RecordTarget.PatientRole.Patient.name.namePart.name [name.entityPartName = “GIV”]	Message_v2–7.SegmentPID.PatientName.GivenName	Patient’s name
ClinicalDocument.RecordTarget. PatientRole.Patient.name.namePart. name [name.entityPartName = “FAM”]	Message_v2–7.SegmentPID.PatientName.FamilyName. Surname	Patient’s surname
ClinicalDocument.RecordTarget. PatientRole.Patient.birthTime	Message_v2–7.SegmentPID.DateTimeOfBirth	Patient’s birth date
ClinicalDocument.RecordTarget. PatientRole.Patient. administrativeGenderCode	Message_v2–7.SegmentPID.AdministrativeSex	Patient’s gender
ClinicalDocument.RecordTarget.PatientRole.Patient.raceCode	Message_v2–7.SegmentPID.Race	Patient’s race
ClinicalDocument.RecordTarget. PatientRole.Patient.maritalStatusCode	Message_v2–7.SegmentPID.MaritalStatus	Patient’s marital status
ClinicalDocument.RecordTarget. PatientRole.Patient.religiousAffiliationCode	Message_v2–7.SegmentPID.Religion	Patient’s religion
ClinicalDocument.RecordTarget. PatientRole.Patient.ethnicGroupCode	Message_v2–7.SegmentPID.EthnicGroup	Patient’s ethnic group

**Fig. 12:**
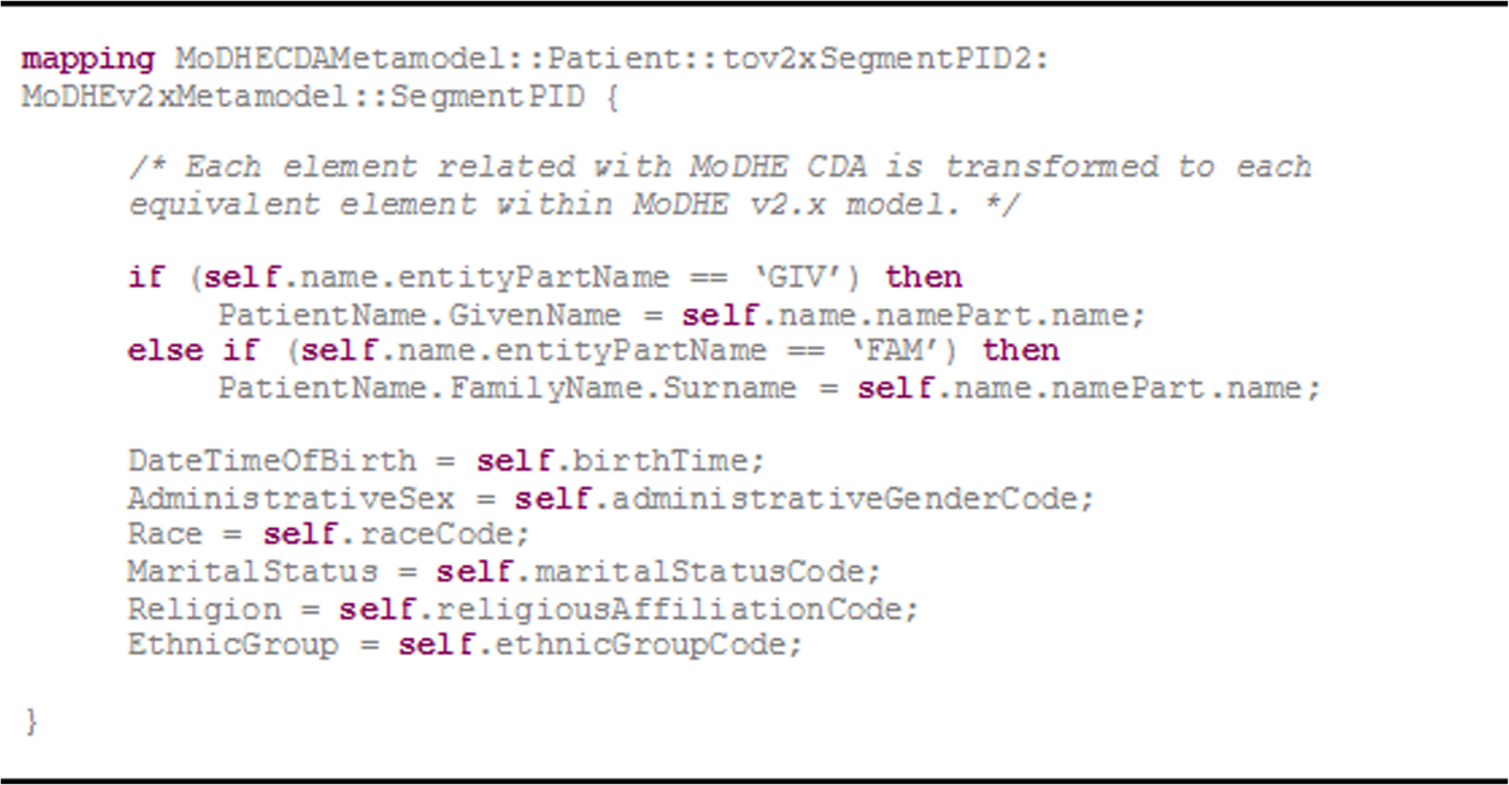
QVT mapping from “Patient” element to “SegmentPID” element

Figure 12 shows the QVT function that maps the eight correspondences included in Table 1.

### MoDHE suite

MoDHE Suite is a tool that supports MoDHE reference framework. It also checks that a practical implementation of the methodology is performed.

As well as MoDHE methodology extends NDT methodology, MoDHE Suite tool uses NDT Suite tool as a base.

NDT Suite is a set of open-source software tools developed to guide the software engineer in the use of NDT methodology. It covers development, quality control, maintenance, test and security. NDT Suite uses and extends UML models following a MDE-based procedure.

With MoDHE metamodel as a reference, which is an abstract syntax of HL7-based model language, a concrete syntax has been defined. Thus, UML profiles have been used with the aim to define new concepts from already existing builders in UML, obtaining a more precise and concrete semantics that is able to model all the existing and varied HL7 standards. We have extended “RA” requirements from NDT to support different HL7 standards. A stereotype in UML profile that extends an UML metaclass is identified from each defined element in MoDHE metamodels. Particularly, all UML profile stereotypes of MoDHE extend UML Class metaclass. This implies that all MoDHE models are designed using the same visual notation as UML classes.

**Fig. 13:**
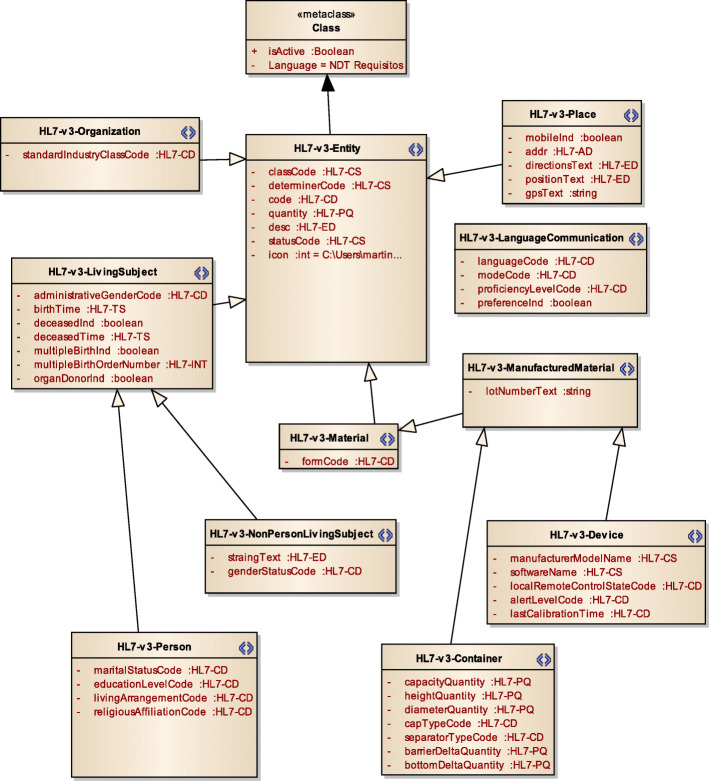
MoDHE UML profile implemented in Enterprise Architect (EA)

Figure 13 shows a subset of MoDHE UML profile, corresponding to some entities of HL7 CDA standard. MoDHE UML profile has not been described in depth, since it is out of the scope of this paper and it would it would become too extensive.

In addition to UML profile developed in EA, we have implemented constraints associated to the model language, transformations among models, as well as user interface developed in C# to provide the software engineer a tool to design domain models according to HL7.

**Fig. 14:**
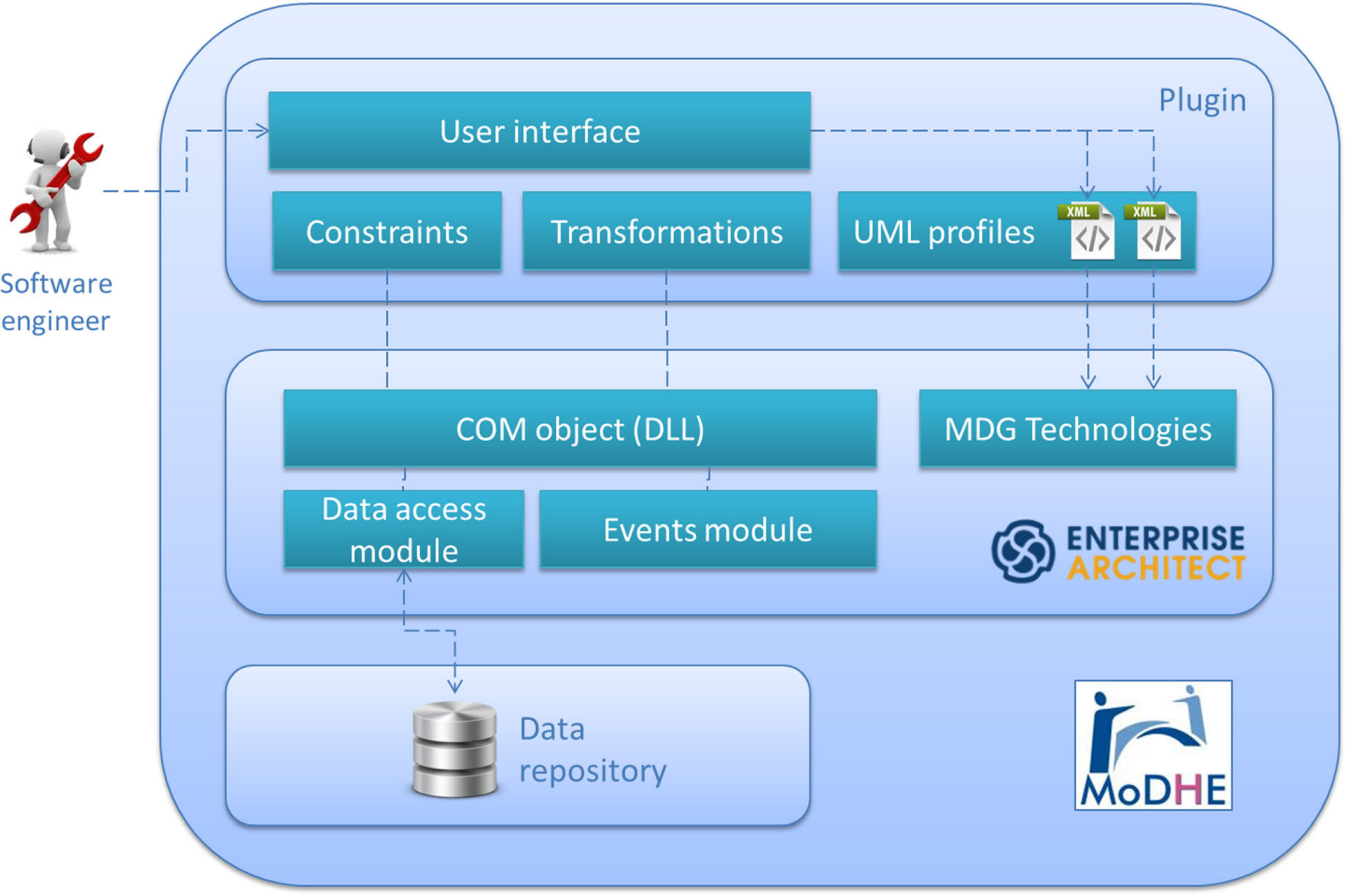
MoDHE Suite architecture

Figure 14 shows the global architecture of MoDHE Suite tool, including UML profile developed in EA together with constraints, transformations and user interface implemented in C# language. The result is a COM object, i.e. a DLL file, that manages the use of constraints and transformations on a set of UML profiles developed with the MDG Technologies of Enterprise Architect.

## Results and validation

MoDHE reference framework and MoDHE Suite tool has been developed for supporting this methodology. Also, a validation process was carried out, on all the offered functionalities, to confirm the tool was behaving as expected and did not present any bug. This validation attended to the results of a research project called Prevensalud. This project develops an interoperability framework that allows exchanging clinical documents among the computer system of the University Hospital “Hospital Virgen del Rocío” in Seville, Spain, and some external systems from arranged bodies.

**Fig. 15:**
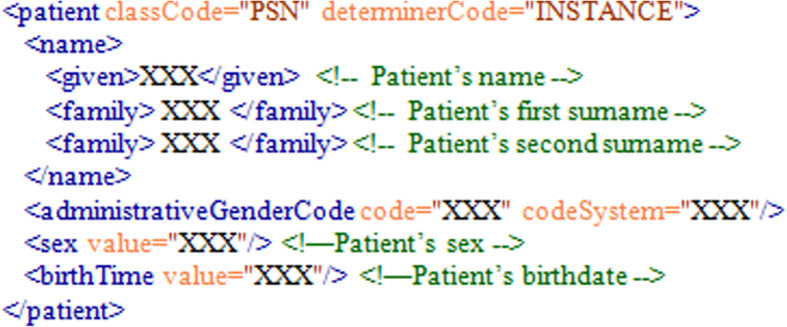
Designing Prevensalud scenario (a part of the CDA scheme)

**Fig. 16:**
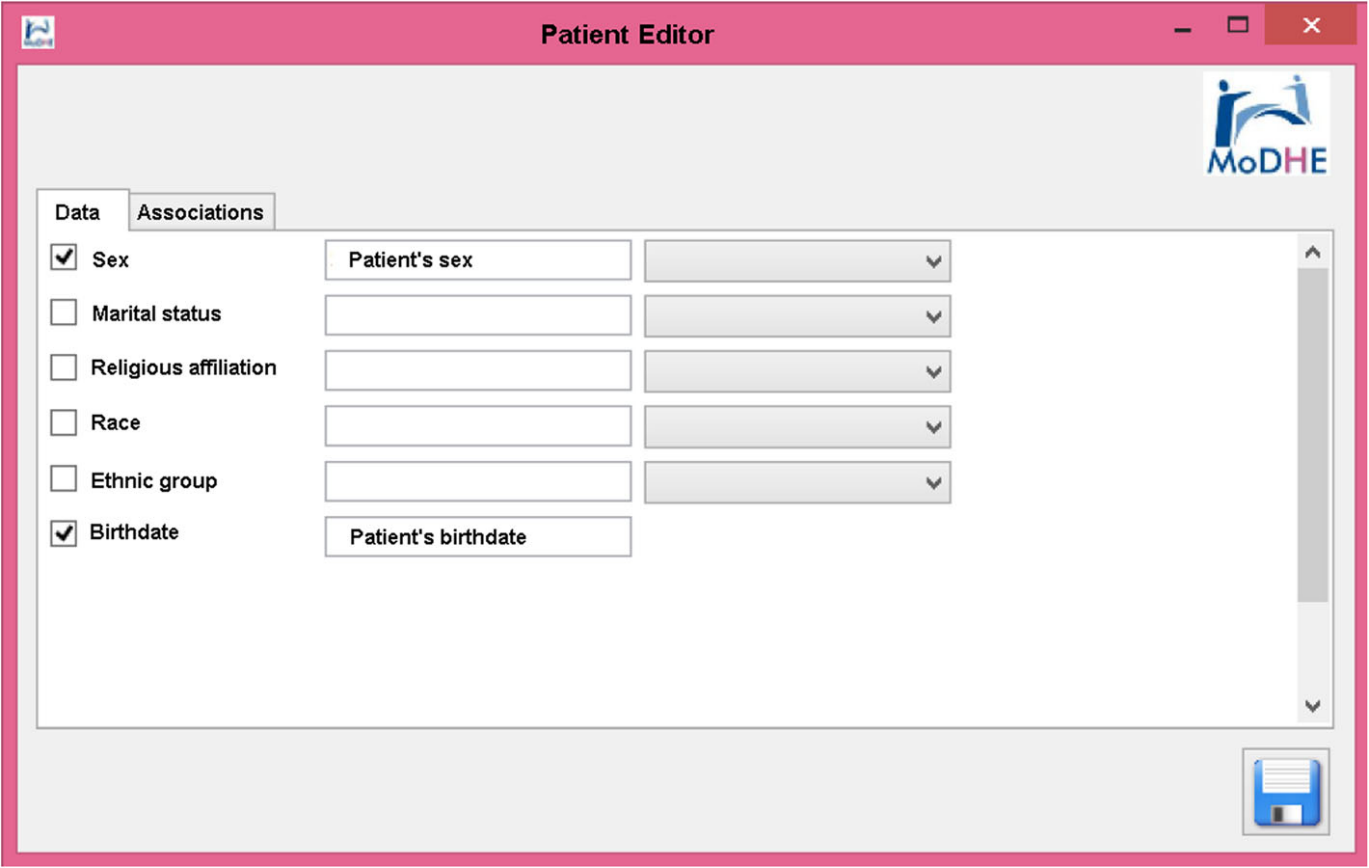
Designing Prevensalud scenario (Data tab in Patient Editor)

**Fig. 17:**
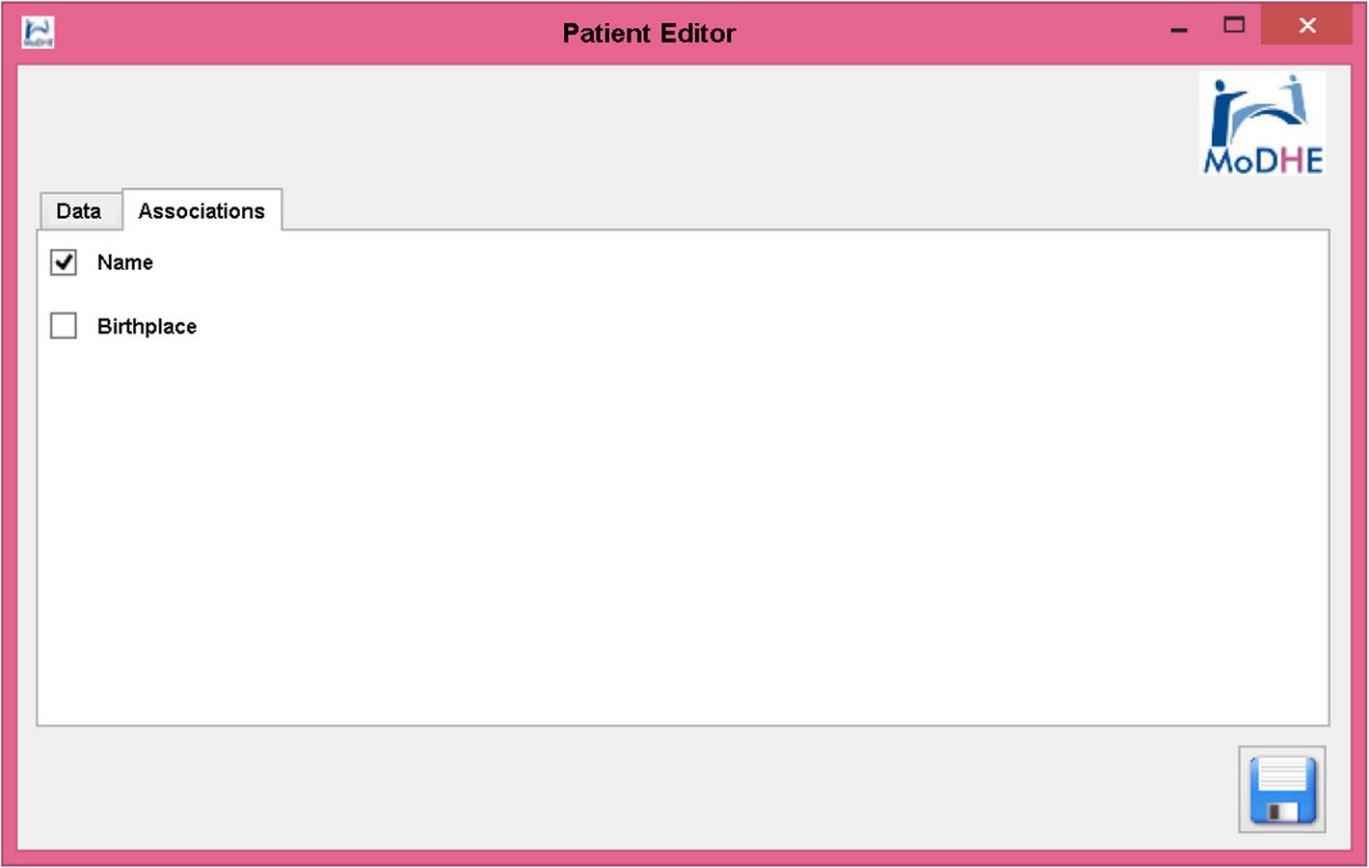
Designing Prevensalud scenario (Associations tab in Patient Editor)

**Fig. 18:**
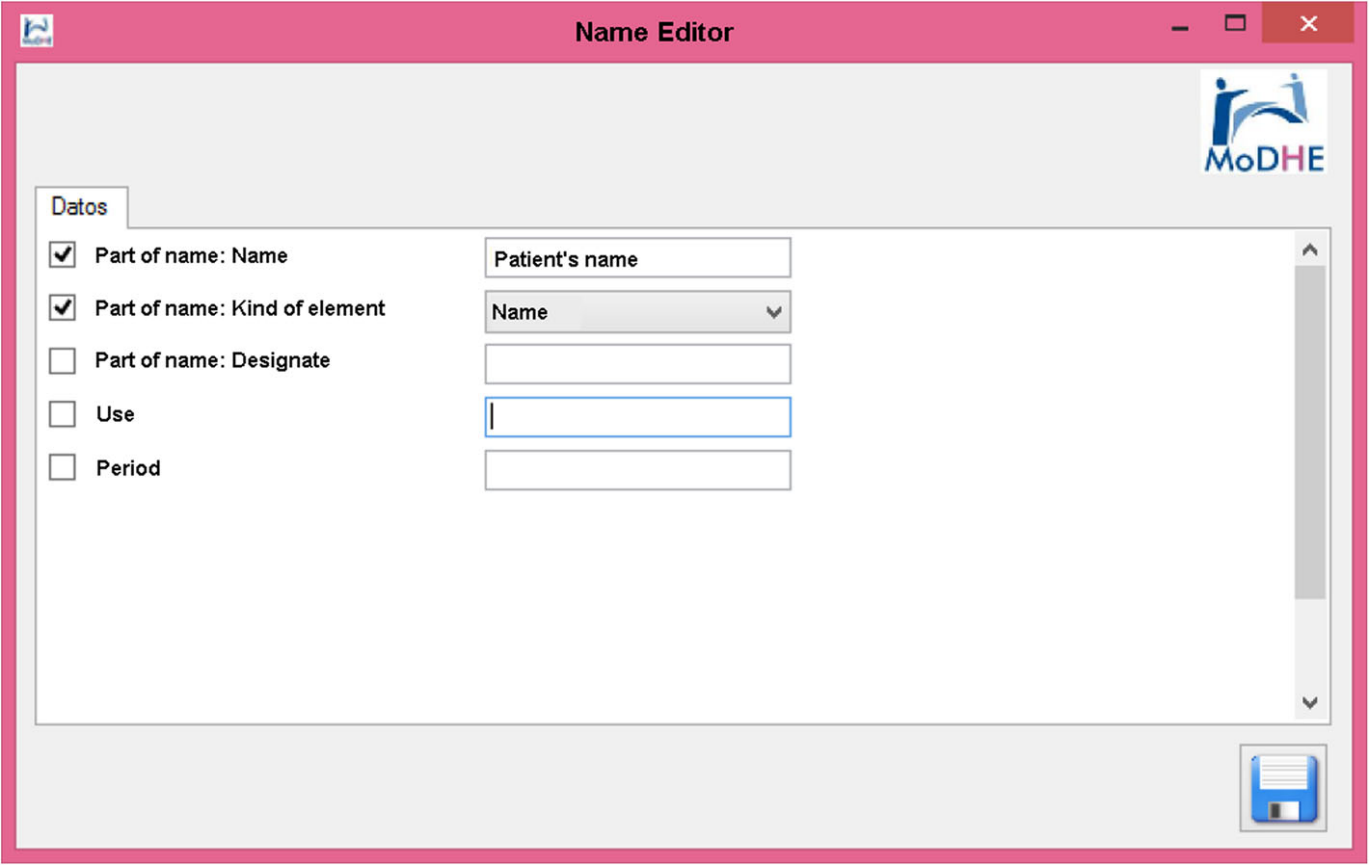
Designing Prevensalud scenario (Name Editor)

Some screenshots from the designing stage of Prevensalud project has been included in Figs. 15, 16, 17 and 18 as example of the user interface at dissimilar stages.

The functional validation performed consisted in designing HL7 models in Prevensalud scenario using the UML interface, that MoDHE Suite tool offers. This validation was carried out by comparing the generated HL7 CDA model with the HL7 CDA model used in the project.

Such comparison uncovered some differences. Therefore, the following step was to review in detail the HL7 CDA standard to verify what model was more consistent with the standard. No errors were found when comparing the model modeled with MoDHE Suite tool with the standard. However, three kinds of errors were identified in the CDA model used in this project. Table 2 lists those errors.

**Table 2:** Diverse kinds of errors

Identifier	Error
Mandatory	Mandatory element in the standard that has not been included in the model.
Value	Element with a mistaken value compared to the standard.
Multiplicity	Element with a maximum multiplicity of 1 in the standard and with multiple multiplicity in the model.

Table 3 shows the errors identified regarding CDA model in the project. They were classified according to the categorization defined in Table 2.

**Table 3:** Identified errors in the CDA model

Class	Attribute	Error identifier and detail
ClinicalDocument	classCode	Mandatory
ClinicalDocument	moodCode	Mandatory
legalAuthenticator	–	Multiplicity
OrganizationPartOf	classCode	Mandatory
OrganizationPartOf	id	Mandatory
AuthoringDevice	classCode	Mandatory
AuthoringDevice	determinerCode	Mandatory
ParentDocument	classCode	Mandatory
ParentDocument	moodCode	Mandatory
inFulfillmentOF	typeCode	Mandatory
Order	classCode	Mandatory
Order	moodCode	Mandatory
ManufacturedProduct	classCode	Mandatory
Material	classCode	Mandatory
Material	determinerCode	Mandatory
consumable	typeCode	Mandatory
product	typeCode	Mandatory
PlayingEntity	determinerCode	Mandatory
PlayingEntity	classCode	Value. The standard defines a mandatory value equal to “ENT”. The implementation guide defines “PLC” or “MMAT”.
participantRole	classCode	Value. The standard defines a mandatory value equal to “ROL”. The implementation guide defines “SDLOC” or “MANU”.

The classCode attribute related to the ClinicalDocument class is shown as an example of a “Mandatory” error. In the Prevensalud CDA guide, this attribute was not included as part of this class, although it is mandatory according to CDA standard.

The legalAuthenticator class is an example of a “Multiplicity” error. In the CDA guide of Prevensalud, the legal authenticator cannot be multiple, since its multiplicity is 0..1. However, Prevensalud CDA offers multiple ability to the legalAuthenticator class.

The classCode attribute related to the participantRole class is given as an example of a “Value” error. In the Prevensalud CDA guide, this attribute is assigned to values “SDLOC” or “MANU”. Nevertheless, the CDA standard defines a mandatory value equal to “ROL” to the classCode attribute as part of the participantRole class.

The result of this validation evinced the fact that developing the HL7 model in the project led to some errors that should have been avoided by using MoDHE Suite tool.

## Discussion and future work

MoDHE reference framework offers to the research community a methodological framework that allows the software engineer to design HL7 domain models by using an UML-based interface, to develop maintainable and adaptable systems, and improves usability and reduces the learning curve.

This study has been developed within the lines of work of two research groups: Web Engineering and Early Testing (IWT2) and Technological Innovation Unit (GIT).

IWT2 successfully presents, as one of the main strategic lines, the combination of the MDE paradigm with information management in numerous contexts, with the aim to cope with the identified needs. Working in a research line dealing with applying the MDE paradigm allows harmonizing proposals and approaches. In consequence, the results of a research work support the hypothesis of subsequent research works, thus progressing jointly. As one of the main goals of this research lines we can point out the need to offer solutions in the health engineering framework. This goal is accomplished in a way that let the software engineer develop solid and maintainable health information systems through the MDE paradigm.

GIT presents, as one of its main strategic lines, the use of health computing standards. The group aims to offer new knowledge to the research community about the real benefit that these standards may provide. In some projects, the members of GIT have been doing some tests with some HL7 standards (v2.x, CDA, FHIR, CCOW, vMR, etc.) and different standards proposed by other standardization organizations (e.g. OpenEHR, ISO 13606 or ISO 13940). Nowadays, HL7 FHIR (Fast Healthcare Interoperability Resources) standard is a particularly important resource to keep in mind in health information systems development. FHIR combines the best characteristics of the most used HL7 standards so far [28–30]. An at once evolution of MoDHE is the inclusion of FHIR, metamodeling the structure of resources that FHIR proposes, allowing to the software engineers develop health information systems based on FHIR without having knowledge about FHIR. If a specific system needs to extend FHIR, only a person in the software engineers’ team must know FHIR deeply to extend the metamodel and to generate the support that the rest of engineers could use without know FHIR. Therefore, the development of MoDHE reference framework has been an important achievement for this research line within GIT research group, as they have offered a robust support to develop HL7 domain models through a methodology based on the MDE paradigm. This way, they have reduced the learning curve to the software engineer who needs to develop HL7 domain models, and they have also generated maintainable and adaptable health information systems, by offering an interface based on UML models.

Furthermore, the present research study has led to new research lines of work that will be detailed in the next subsections.

### Align HL7 standards with a standard system of concepts

There are standard systems of concepts in the health context, such as ISO 13940 standard, which define the list of concepts that could be identified in a specific scenario. Aligning the concepts used in HL7 standards with this kind of system of concepts is a complex and tedious task.

However, as MoDHE reference model has been developed following a methodology based on MDE, relations between concepts used in HL7-based model languages, and those included in the system of concepts, could be identified. Therefore, they would play the role of aligning the concepts used in HL7, as well as in the system of concepts in a more simple and maintainable way.

The Alignment concepts included in HL7 standards and in a system of concepts is a complementary research line, that could be further developed in the future.

In addition, an online, public and complete version of MoDHE will be published to make it available to the scientific community as complementary material.

### Certify the conformance of existent HL7 models

For years, lots of health information systems were developed designing HL7-based domain models, without any supporting methodology that guarantee conformity of these domain models in the use of HL7 standards.

An interesting research work, that appears from the present research line, is the lack of mechanisms for existent HL7-based domain models to certify their conformity in the use of HL7 standards.

It would be interesting for HL7 to have a methodology that enables validating a domain model design, using the rules defined in at least one HL7 standard. This method could begin by designing a certification process for current HL7-based health information systems.

## Conclusions

The present research line has been developed with the challenge to help software engineers’ community design domain models in UML conforming to HL7, using the MDE paradigm. The pursued scenario is to approach domain models of HL7-based health information systems with UML design, focusing on the richness and wide use that UML entails within that community.

The present methodology proposes to support the design of HL7 domain models using UML models through a model-driven solution. Therefore, a software engineer without any knowledge about HL7 standards can model the project’s requirements as well as HL7 standards rules, its recommendations, and constraints for HL7-based health information systems by using UML elements. It is intended to distribute the produced framework by making it available for the HL7 community from the material published in our PhD Thesis [31].

## Data Availability

Not applicable.

## Abbreviations

UML: Unified Modeling Language
OMG: Object Management Group
MDE: Model-Driven Engineering
HL7: Health Level 7
MIF: Model Interchange Format
NDT: Navigational Development Techniques
MoDHE: Model-Driven Health Engineering
DRS: System Requirement Development
RA: Information Requirements
QVT: Query/View/Transformation
EA: Enterprise Architect
IWT2: Web Engineering and Early Testing research group
GIT: Technological Innovation Unit research group
FHIR: Fast Healthcare Interoperability Resources
